# Possible germ cell-Sertoli cell interactions are critical for establishing appropriate expression levels for the Sertoli cell-specific MicroRNA, miR-202-5p, in human testis

**DOI:** 10.1186/s12610-015-0018-z

**Published:** 2015-03-03

**Authors:** Ali A Dabaja, Anna Mielnik, Brian D Robinson, Matthew S Wosnitzer, Peter N Schlegel, Darius A Paduch

**Affiliations:** Department of Reproductive Medicine, Weill Cornell Medical College, 525 East 68th St Starr 900, New York, NY 10065 USA; Department of Pathology, Weill Cornell Medical College, 522 East 68th St. Starr 100, New York, NY 10065 USA

**Keywords:** MicroRNA, Spermatogenesis, Male infertility, miR-202-5p, Sertoli cells, microARN, Spermatogenèse, Infécondité masculine, miR-202-5p, Cellules de Sertoli

## Abstract

**Background:**

To examine human microRNA expression in fertile men and subsequently to compare expression patterns of miRNAs in fertile and infertile men, specifically men with Sertoli Cell Only (SCO) histopathology.

**Methods:**

Testicular tissues from men with azoospermia and SCO, as well as those of men with normal spermatogenesis, were analyzed. MicroRNA was isolated using the miRCURY™ RNA Purification Kit. A miRCURY LNA™ Universal RT system was used for detection of microRNA by quantitative real-time PCR. MicroRNA localization was performed by in situ hybridizations (ISH) on formalin-fixed paraffin embedded (FFPE) tissue utilizing miRCURY LNA™ microRNA ISH technology. Statistical analysis was performed by GenEx V5.0.

**Results:**

MicroRNA expression was determined for 13 normal fertile men and 5 men with the confirmed diagnosis of diffuse SCO. MiR-202-5p expression was reduced by 17-fold (P < 0.00001) in tissue from SCO men compared to normal. MiR-34c-5p was reduced by 346-fold (P < 0.00001), miR-10b was reduced 18-fold (P < 0.00001), miR-191 was reduced 20-fold (P = 0.001) and miR-126 was reduced 40-fold (P < 0.00001)) in tissues from SCO compared to normal fertile men. Using ISH, miR-202-5p was localized to Sertoli cells of men with normal spermatogenesis, but not in the Sertoli cells of men with SCO.

**Conclusion:**

Number of miRNAs are differentially expressed in normal fertile men compared to men with SCO. MicroRNA-202-5p is localized to Sertoli cells and its expression dramatically differs between fertile men and men whose germ cells are depleted, suggesting a novel interaction for regulating microRNA expression between the somatic and germ cell components of the seminiferous epithelium.

## Abstract (fr)

**Objectifs:**

Evaluer l’expression des microARN chez des hommes féconds puis comparer les profils d’expression de ces miRNAs chez des hommes féconds et des inféconds qui présentent plus particulièrement un syndrome de Sertoli seules (SCO) à l’histologie testiculaire.

**Matériel et Méthodes:**

Ont été analysés des tissues testiculaires d’hommes avec azoospermie et SCO ainsi que ceux d’hommes avec spermatogenèse normale. Les miRNAs ont été isolés avec la trousse de Purification miRCURY™ RNA. Le système miRCURY LNA™ Universal RT a été utilisé pour la détection quantitative de miARNs par PCR en temps réel. La localisation des miARNs a été réalisée par hybridation in situ (HIS) sur des tissus fixés au formol et inclus en paraffine en utilisant la technologie miRCURY LNA™ microRNA ISH. Les analyses statistiques ont été faites avec GenEx V5.0.

**Résultats:**

L’expression des microARNs a été faite chez 13 hommes féconds et 5 hommes avec un diagnostic confirmé de SCO diffus. L’expression de miR-202-5p est réduite d’un facteur 17 (P < 0.00001) dans le tissu des hommes SCO par rapport au tissu des hommes à spermatogenèse normale. L’expression de miR-34c-5p est réduite d’un facteur 346 (P < 0.00001), celle de miR-10b d’un facteur 18 (P < 0.00001), celle de miR-191 d’un facteur 20 (P = 0.001) et celle de miR-126 d’un facteur 40 (P < 0.00001) dans les tissus des hommes SCO comparés à ceux des hommes à spermatogenèse normale. MiR-202-5p a été localisé par HIS dans les cellules de Sertoli des hommes à spermatogenèse normale, mais pas dans les cellules de Sertoli des hommes SCO.

**Conclusions:**

Nombre de miARNs sont exprimés différentiellement chez les hommes féconds par rapport aux hommes SCO. MicroARN-202-5p est localisé dans les cellules de Sertoli et son expression diffère de façon marquée entre les hommes féconds et ceux dont les cellules germinales sont absentes; ceci suggère une nouvelle interaction – entre les cellules somatiques et germinales constitutives de l’épithélium séminifère – impliquée dans la régulation de l’expression des microARNs.

## Background

Infertility affects 10%–15% of couples worldwide (WHO, 1983) [1]. Half of all infertility cases are due to male factors, and about 60–75% of male infertility is idiopathic. Most “idiopathic” male infertility is thought to be caused by yet-to-be-identified genetic defects [2]. Spermatogenesis is a multistep complex process that displays strictly a regulated spatiotemporal gene expression, and during certain aspects of germ cell division, mRNA translation is significantly repressed [3]. Studies have indicated that microRNAs (miRNAs) may play a role in translational repression during spermatogenesis [4]. Therefore the deregulation of miRNAs could play an essential role in spermatogenic dysfunction.

miRNAs are 20 to 30 nucleotide noncoding single strand RNA molecules that act to regulate mRNA stability, and translation. They interact with their mRNA target through base-pairing, generally in their 3’UTR [5]. miRNAs appear to be evolutionarily conserved and play critical roles in a variety of biological processes in different cell types. Some miRNA show a tissue-specific expression, and several experiments have confirmed their importance in regulating cellular growth and differentiation [5,6]. Moreover, the overexpression of a tissue-specific miRNA in nonrelated cells shifts its transcriptome toward that of the lineage expressing the miRNA, making them a possible target for therapeutic use [7].

The majority of published literature on miRNAs focuses on the role they play in biological processes, including cell proliferation, differentiation, cell growth, death, and resistance to stress [8,9]. Differences in expression profiles of miRNAs have been linked to cancer, heart disease and male infertility [10-13]. However, despite intensive investigations, the cell type specificity of miRNA expression remains poorly understood. In this study we quantitatively examined miRNAs expression in normal human testis and in men with a severe form of infertility: azoospermia associated with Sertoli Cell Only (SCO) syndrome. We also localized miRNAs of interest in testicular tissue to guide a greater understanding of their potential role in spermatogenesis.

## Methods

### Testicular tissues

Weill Cornell Medical College Institutional review board approval was obtained for this study. Testicular tissue for SCO were obtained from male infertility patients during therapeutically necessary procedures and were snap frozen at the time of procurement, or sent to create formalin-fixed paraffin-embedded (FFPE) sections. These men were azoospermic, had Sertoli cell-only documented on testis biopsy, and they had SCO documented in all dissected regions of the testis using a microsurgical approach for testis evaluation as part of a therapeutic attempt at sperm retrieval. Normal testicular tissue was obtained from men with obstructive azoospermia as well as from testes of organ donors utilizing an approved procurement protocol. Written informed consent was obtained from the patient for the publication of this report and any accompanying images.

### RNA isolation

Total RNA was isolated from testicular tissue using the miRCURY™ RNA Isolation Kit (Cat No. 300111, Exiqon Inc. Vedbaek, Denmark). It is based on spin column chromatography using a proprietary resin as the separation matrix. This method of purification of total RNA allows for the isolation of the very small (<200 nucleotide) RNA fraction.

In brief, the frozen tissue is completely homogenized in a lysis solution using TissueRuptor. The lysate is incubated with Proteinase K for protein removal and loaded on a spin column. The removal of genomic DNA is performed directly on the column with DNase I (Qiagen, Venlo, Limburg) at a final concentration of 0.25 Kunitz unit/μL. Eluted total RNA was either used for cDNA synthesis or stored in – 80° Celsius. RNA concentration was measured with a fluorescence based quantitation assay using Qbit® fluorometer (Life Technology™, NY, USA), and the integrity of RNA was evaluated by calculating RNA integrity number (RIN, based on the detection of 18S and 28S and the amount of degradation products) on Agilent Bioanalyzer 2100 (Aligent Technologies, CA, USA). Only RNAs with RIN greater to or equal to 7 were used in the expression studies, this indicate that the RNA sample has minimal degradation products.

### Design of custom Pick-&-Mix testis specific miRNAs panels

Nineteen miRNAs that had been previously reported to be highly expressed in the testis were chosen to design a custom made Pick and Mix testis specific miRNA human panel [14]. Additionally, 3 miRNAs (miR-103, miR-191 and miR-423-5p) with stable expression in human testis tissue were included to serve as reference miRNAs in the expression analysis. Each plate is designed to accept 22 × 4 samples in a pre-defined 96-well panel format to screen 4 different RNAs in one setting. Two wells for each sample were used for inter-plate calibrator (annotated as UniSp3 IPC) to aid with performing plate-to-plate and run-to-run normalization analysis. The miRNAs panels were ordered and prepared from Exiqon Inc. (Vedbaek, Denmark). miRNA names, target sequences and their catalog numbers are listed in (Table 1).

**Table 1:** **miRNA’s names, target sequences and catalog numbers**

**Human miRNA**	**Target sequence**	**Product Cat. No**
hsa-miR-10b-3p	ACAGAUUCGAUUCUAGGGGAAU	204514
hsa-miR-10b	UACCCUGUAGAACCGAAUUUGUG	204753
hsa-miR-34c-3p	AAUCACUAACCACACGGCCAGG	204373
hsa-miR-34c-5p	AGGCAGUGUAGUUAGCUGAUUGC	204407
hsa-miR-99b-3p	CAAGCUCGUGUCUGUGGGUCCG	204064
hsa-miR-99b	CACCCGUAGAACCGACCUUGCG	204367
hsa-miR-125a-3p	ACAGGUGAGGUUCUUGGGAGCC	204446
hsa-miR-125a-5p	UCCCUGAGACCCUUUAACCUGUGA	204339
hsa-miR-126-5p	CAUUAUUACUUUUGGUACGCG	204584
hsa-miR-126	UCGUACCGUGAGUAAUAAUGCG	204227
hsa-miR-202-5p	UUCCUAUGCAUAUACUUCUUUG	204730
hsa-miR-202	AGAGGUAUAGGGCAUGGGAA	204101
hsa-miR-204	UUCCCUUUGUCAUCCUAUGCCU	204507
hsa-miR-506	UAAGGCACCCUUCUGAGUAGA	204539
hsa-miR-508-3p	UGAUUGUAGCCUUUUGGAGUAGA	204480
hsa-miR-508-5p	UACUCCAGAGGGCGUCACUCAUG	204077
hsa-miR-509-3p	UGAUUGGUACGUCUGUGGGUAG	204458
hsa-miR-509-3-5p	UACUGCAGACGUGGCAAUCAUG	204503
hsa-miR-514	AUUGACACUUCUGUGAGUAGA	204645
hsa-miR-103	AGCAGCAUUGUACAGGGCUAUGA	204063
hsa-miR-191	CAACGGAAUCCCAAAAGCAGCUG	204306
hsa-miR-423-5p	UGAGGGGCAGAGAGCGAGACUUU	204593

### cDNA synthesis and real-time qPCR

First strand of cDNA was synthesized using a Universal cDNA Synthesis Kit II (Exiqon, Cat. No 203301). An artificial RNA (kit based RNA spike-in) was added to each reverse transcription reaction as a control to confirm that the reverse transcription and amplification occurred with equal efficiency in all samples. Twenty nanogram of total RNA in 4 μL volume was mixed with 16 μL reverse transcriptase (RT) master mix containing reaction buffer. Enzyme mix and spike-in were incubated in a thermocycler at 42° Celsius for 60 min. RT was then heat-inactivated at 95° Celsius for 5 min and the reactions were cooled down and stored in – 20° Celsius. Negative controls excluding template from the reverse transcription reaction were included and profiled as for the test samples.

The levels of miRNAs in whole testis samples was measured using real-time PCR with custom Pick-and-Mix testis-specific panels and SYBR® Green master mix from Exiqon, (Cat No. 203450). Each plate was pre-loaded with dried out miRNAs in all wells, cDNA from each patient was diluted 1:100 and combined with 2× SYBR® Green master (1:1). Samples and controls were adjusted to 10 μL volume in all 24 wells. The amplification reactions were performed on a LightCycler 480 Roche platform (Roche, IN, USA). The samples were subjected to 95° Celsius denaturation for 10 min followed by 45 cycles of 95° Celsius for 10 sec and 60° Celsius for 1 min with optical read at the end of every cycle. Melting curve analysis was carried out to check for examination of amplification products.

### In Situ Hybridization (ISH)

To localize the differentially expressed miRNAs from testicular tissues (to determine the cellular origin and evaluate relative expression levels) ISH was performed on FFPE sections. Double-Dig-labeled probe for miR-202-5p (miRCURY LNA™ Detection probe, 5′-DIG and 3′-DIG labeled hsa-miR-202-5p, Exiqon, Cat No. 38814–15) was used. As a negative control, a scramble probe was used (Probe No. 90–001). Additionally, as positive control for testicular tissue, the has-miR-126 probe for endothelial cells was tested (Probe No. 90–008). ISH was performed using manufacturers instructions. In brief, all FFPE sections were deparaffinized in two washes of CitriSolve for 15 min and then hydrated through sequentially increasing ethanol solutions. The target demasking step was carried out in Proteinase K solution at a concentration of 15 μg/mL at 37° Celsius for 10 min. Following pre-digestion that allows the access of double-DIG-labeled LNA™ probes to hybridize to the miRNA sequence, sections were dehydrated in gradual ethanol solutions and dried completely for 20 min. Probe, denatured at 90° Celsius for 4 min, was diluted in 1x hybridization solution to a final concentration of 40nM. A total of 50 μL was applied on each slide. The slides were then covered with cover glass, sealed with Fixogum and hybridized for 60 min. To optimize performance for each probe and to ensure optimal signal to noise ratio, hybridization was performed at a temperature of 30° Celsius below the probe melting temperature. The slides were passed through stringent washing steps of 5X, 1X and 0.2X SSC at hybridization temperature. Digoxin was recognized by a sheep anti-DIG-AP directly conjugated with the enzyme Alkaline Phosphatase (AP) (Roche, Cat. No. 11 093 274 910). The specimens were incubated for 60 min at room temperature. AP converted the applied substrate NBT/BCIP (Roche, Cat. No. 11 697 471 001) to the soluble substrates 4-Nitro-Blue Tetrazolium (NBT) and 5-Bromo-4-Chloro-3’-Indolylphosphate (BCIP) into a water and alcohol insoluble dark-blue NBT-BCIP precipitate that appears on slides after 2 h of incubation at 30° Celsius in the dark. The reaction was stopped and slides were counterstained with filtered Nuclear Fast Red™ (Vector laboratories, Cat. No. H-3403). After washing in tap water for 10 min and dehydration with ethanol, the slides were mounted using Eukitt® mounting media (VWR, Cat. No. 361894G). The slides were examined by light microscopy the subsequent day and images were obtained using Kodak microscope.

### Statistical analysis

#### Analysis of miRNA

The expression level of miRNAs was performed based on absolute quantification analysis using second derivative method by LightCycler 480 software from Roche allowing Cp values to be calculated. GenEX software (MultiD Analyses AB, Göteborg, Sweden) was used to analyze and normalize the miRNA-qPCR data. This program allows the correction of PCR efficiencies, the compensation for differences between runs by normalizing with interplate calibrators, and normalization with endogenous reference genes. Data was normalized to PCR efficiency (E), $CpE=100\%=CpE\frac{ \log \left(1+E\right)}{ \log 2}$, and for differences between runs using Interplate normalisation and Interplate control (IPC): $Cpnormal=Cp-\frac{1}{n}{\displaystyle \sum_{i=1}^n CpIC}$. The Cp values of genes of interest were normalized to plate average after removing Cp values that were greater than 38. Normalisation of the gene of interest (GOI) to reference Cp was done using the following formula: $CpGOI, norm= CpGOI-\frac{1}{n}{\displaystyle \sum_{i=1}^n Cpavg}$. SCO was further normalized to the relative expression of the same miRNA in normal tissue to determine the fold changes of differential expression in SCO tissue. The formula for normalisation of SCO to normal tissues was the *RatioT*/*N* = 2^− *CP*(*SCO*) + *Cp*(*Normal*)^. The normality distribution of the data was tested by the Kolmogorov-Smirnov test. Parametric analysis (*t*-test according to Student with paired and unpaired values) was used as appropriate. P-values below 0.05 were regarded as statistically significant.

## Results

Expression of miRNA was determined for 13 normal fertile men and 5 men with the confirmed diagnosis of diffuse SCO using the 96 well plates of previously selected primers that are relevant for testicular tissue. Mean age of the cohort was 35(SD ± 6.7) years old. By applying an unpaired two-tailed *t*-test for miRNAs that showed >2-fold change in the considered groups, we found 8out of the 22 miRNA with significant differences in expression levels when comparing the samples from SCO samples with the normal testicular samples (P < .005). Detail of miRNA expression for SCO samples compared to normal are listed in (Table 2). This is consistent with the fact that the greatest changes detected in SCO/Normal miRNA comparisons were for miR-34c-5p that was downregulated by a factor of 346 (P < 0.00001). mir-34c-5p is believed to be predominantly expressed in germ cells [10], reflecting the SCO miRNA expression pattern in our samples. miR-126 was downregulated by a factor of 40 (P < 0.00001), miR-126-5p by a factor of 20.7, miR-191 by a factor of 20 (P = 0.001) in SCO samples relative to normal testicular tissue. miR-202-5p expression was reduced by 17-fold (P < 0.00001) in SCO men compared to normal. No significant increase in miRNA expression levels was seen in men with SCO relative to normal testicular tissue levels.

**Table 2:** **miRNA expression profiling in normal vs. SCO**

miRNA	Normal vs. SCO	P-Value
hsa-miR-34c-5p	−346	<0.00001
hsa-miR-126	−40	<0.00001
hsa-miR-191	−20	<0.001
hsa-miR-126-5p	−20.7	<0.001
hsa-miR-10b	−18	<0.0001
hsa-miR-202-5p	−17	<0.00001
hsa-miR-103	−7	0.001
hsa-miR-514	−5.8	0.01
hsa-miR-509-3-5p	−4.2	0.007
hsa-miR-204	−4.2	<0.001
hsa-miR-10b-3p	−3.3	0.06
hsa-miR-508-3p	−3	0.04
hsa-miR-125a-5p	−2	0.02
hsa-miR-99b-3p	−2.4	0.008
hsa-miR-99b	−2	0.008
hsa-miR-34c-3p	−2.3	0.06
hsa-miR-508-5p	−2	0.13
hsa-miR-506	−2	0.14
hsa-miR-125a-3p	−1	0.40
hsa-miR-509-3p	1	0.45
UniSp6 CP	1	0.34
hsa-miR-423-5p	1.5	0.11
hsa-miR-202	1.6	0.10

**Figure 1:**
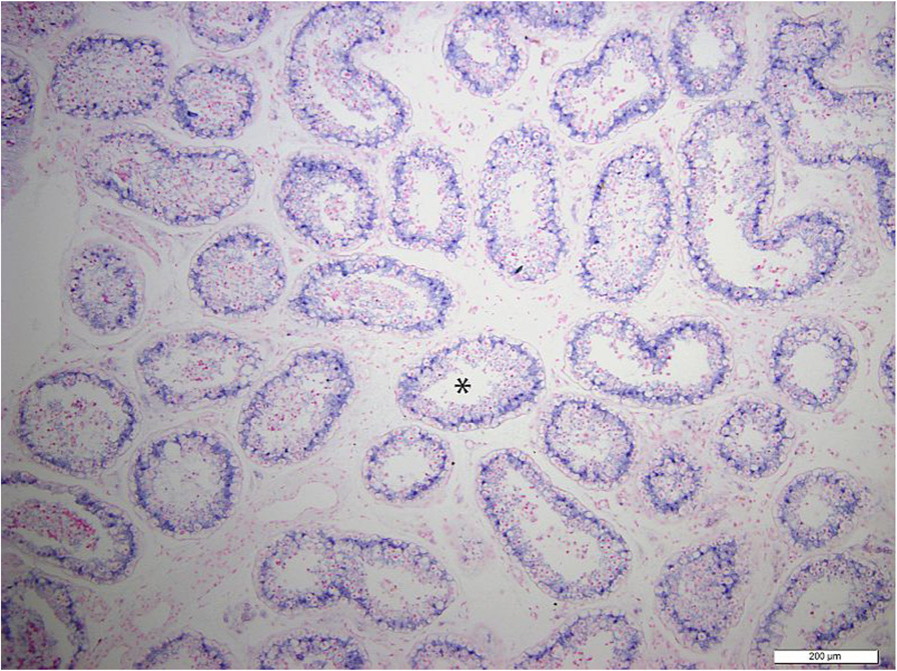
ISH of miR-202-5p, blue precipitate indicate cellular expression of miRNA in normal testicular tissue, *normal seminiferous tubules, scale 200 μm.

**Figure 2:**
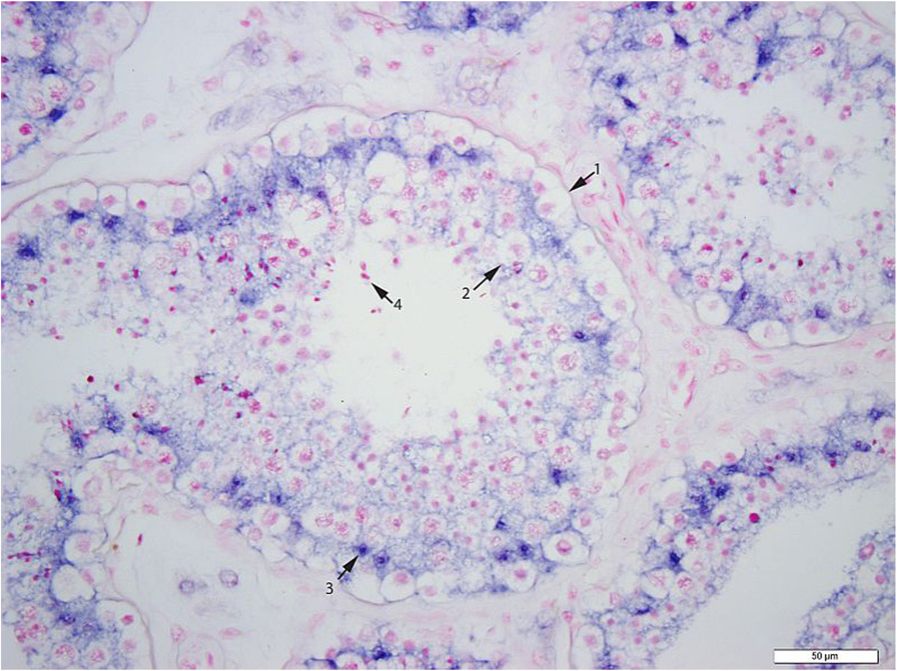
**ISH of miR-202-5p, blue precipitate indicate cellular expression of miRNA in normal testicular tissue, 1 spermatogonia, 2 primary spermatocyte, 3 Sertoli cell, 4 spermatid, scale 50 μm.**

**Figure 3:**
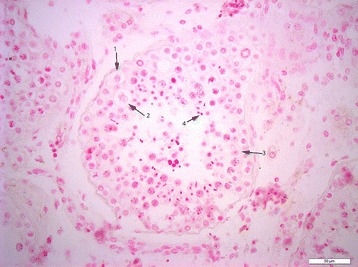
**ISH of negative control in normal testicular tissue, 1 spermatogonia, 2 primary spermatocyte, 3 Sertoli cell, 4 spermatid, scale 50 μm.**

**Figure 4:**
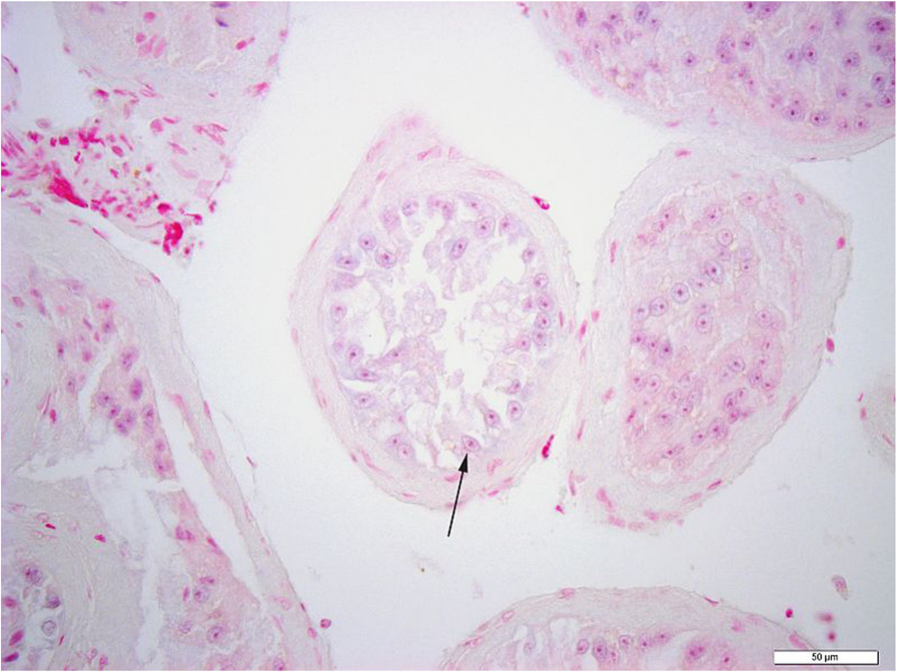
**ISH miR-202-5p in testicular tissue of men with SCO, arrow Sertoli cells, scale 50 μm.**

**Figure 5:**
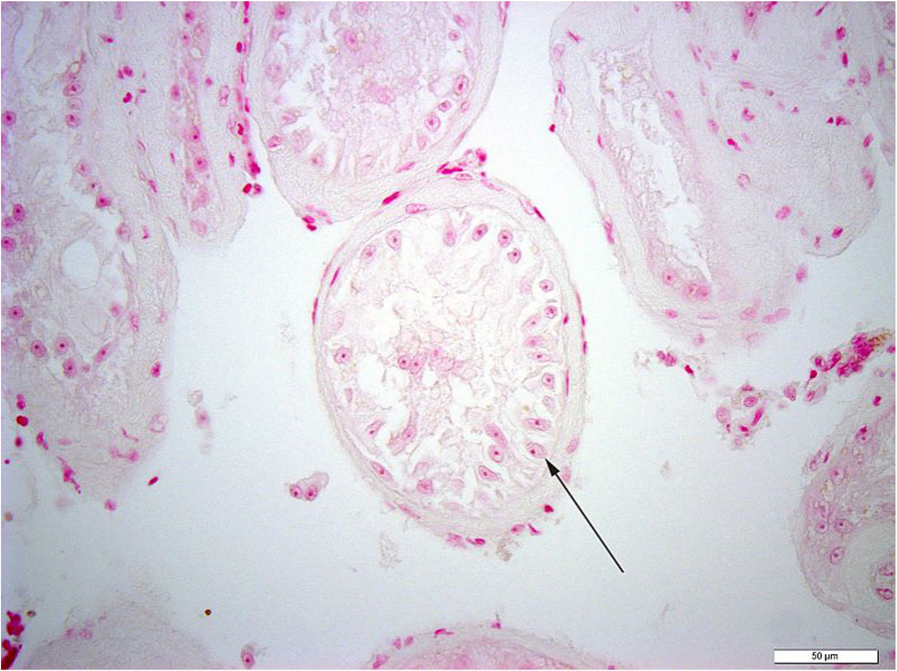
**ISH negative control in testicular tissue of men with SCO, arrow Sertoli cells, scale 50 μm.**

To further decipher the expression profile in testicular tissue, and to determine the cell types that express miR-202-5p, ISH was performed on both normal and SCO testicular tissue. Hybridization signal specific for of miR-202-5p was specifically detected in the Sertoli cells of men with normal spermatogenesis (Figures 1, 2 and 3). The tissue of men with confirmed SCO had no signal detectable for miR202-5p in Sertoli cells (Figures 4 and 5). These observations reflect that miR-202-5p are selectively expressed in Sertoli cells in men with normal germ cells, but not in the Sertoli cells of men with SCO. This localization pattern of miR-202-5P is consistent with our qPCR results. There were no differences in expression of control miRNAs between men with normal spermatogenesis and SCO, using scramble for negative control and miR-126 specific to endothelial as a positive control.

## Discussion

Profiling of miRNA in human and murine testis has previously been reported in multiple published studies [14-16]. The testis specific expression of miRNAs in normal men and men with clinical infertility have been corroborated by these studies as well as the data in this manuscript. Several miRNA clusters and their target genes as well as their chromosomal origin have also been examined [14,15]. However, the cellular localization of specific testicular miRNAs have not been well elucidated in human testicular tissue. Since azoospermic men with failed spermatogenesis have pathology ranging from Sertoli cell-only pattern to maturation arrest, or hypospermatogenesis, analysis of the cellular origins of these differentially expressed miRNAs is important to better reflect their potential regulatory roles in spermatogenesis and male infertility. In our study we used ISH to localize miRNA expression. ISH has been previously shown to reveal cellular localization of mRNAs and non-coding RNA [17].

Several of the testicular specific miRNAs identified in our study have been evaluated by previous studies. However, we identified eight novel miRNAs that have not been detected previously and may play a role in testicular function and spermatogenesis. We found that miR-204, miR-99b-3p, miR-191, miR-509-3-5p, miR-99b, miR-10b, miR-126, miR-126-5p, miR-103 are downregulated in samples from men with SCO pathology pattern. We could only identify three prior reports on the use of ISH to localize miRNA to specific cell types in testes. ISH of the miR-34 family indicated that miR-34b/c are primarily localized to spermatocytes and spermatids in murine testicular tissue [15]. Whereas miR-34a appeared to be restricted to murine spermatogonia residing on the basal membrane of seminiferous tubules [18]. In another study, miR-383 was highly expressed in primary spermatocyte and poorly expressed in spermatids of samples of men with normal spermatogenesis; whereas the expression of miR-383 in tissues from men with severely impaired sperm production was noticeably lower compared with the control group [13]. Our study demonstrates that miR-202-5p is selectively expressed in Sertoli cells in men with normal germ cells, but not in the Sertoli cells of men with SCO.

To date, no direct physiological function for miR-202-5p has been identified in humans. miR-202-5p/3p are a member of the let-7 family. let-7 was the first human miRNA to be discovered. let-7 and its family members are highly conserved across species in sequence and function. Deregulation of let-7 leads to a less differentiated cellular state and the development of cell-based diseases. In general high levels of let-7 expression is associated with cell differentiation and maturation [19]. miR-202-5p is expressed in Sertoli cells in the early XY gonad [20]. Its expression was found to be downstream of the testis-determining factor SOX9, that plays an important role in testicular development and maturation [21]. Previous studies have suggested that miR-202 expression increases in testicular tissue postnatally and during development, suggesting that the expression of miR-202-5p is up-regulated in the testis at later developmental stages [14]. These data indicate that miR-202-5p expression is associated with male gonad development, and its expression is restricted to Sertoli cells in the adult testis.

The pathophysiology that leads to SCO pathology pattern is not completely understood. It is debatable if Sertoli cells did not develop normally so that germ cells could not survive, or the function of Sertoli cells is normal, but SCO has arisen because of an inherent problem with the germ cells. Sertoli cell dysfunction secondary to the absence of other testicular cell types can occur [22,23]. The absence of germ cells can result in de-differentiation of Sertoli cells, so that Sertoli cell function reflects that of immature Sertoli cells, leading to loss of expression of miR-202-5p expression. Conversely, it is important to recognize that the absence of germ cells may also be a reflection of underlying abnormalities in the Sertoli cells and failure of their maturation [24]. During puberty, Sertoli cells undergo a radical change in their morphology and function, reflecting maturation from an immature proliferative state to a non-proliferative state [25]. This maturation process is highly dependent on miRNAs, studies have shown that Dicer-mutant Sertoli cells show a progressively aberrant development and loss of function [26]. The Sertoli cell maturation process switches on multiple cell functions. Previous studies have suggested that miR-202 expression increases in testicular tissue postnatally and during development, indicating that the expression of miR-202-5p is up regulated in the testis at later stages during development [14]. Further rodent studies may help elucidate whether loss of miR-202-5p is the cause or a consequence of loss of germ cells in men with Sertoli Cell Only syndrome.

## Conclusion

Our results reveal several miRNAs that are differentially expressed in normal fertile men compared to men with SCO. miR-202-5p is highly expressed in Sertoli cells when germ cells are present. This germ cell-dependent expression of miR-202-5p suggests a functional role of this miRNA in Sertoli cell maturation and/or regulation of spermatogenesis.

## Abbreviation

SCO: Sertoli Cell Only
ISH: In Situ Hybridizations
FFPE: Formalin-Fixed Paraffin Embedded
miRNAs: microRNAs
ISH: In Situ Hybridization
